# FAZ assembly in bloodstream form *Trypanosoma brucei* requires kinesin KIN-E

**DOI:** 10.1091/mbc.E23-01-0022

**Published:** 2023-08-17

**Authors:** Anna C. Albisetti, Robert L. Douglas, Matthew D. Welch

**Affiliations:** a Department of Molecular and Cell Biology, University of California, Berkeley, CA 94720; Chinese Academy of Sciences

## Abstract

*Trypanosoma brucei*, the causative agent of African sleeping sickness, uses its flagellum for movement, cell division, and signaling. The flagellum is anchored to the cell body membrane *via* the flagellum attachment zone (FAZ), a complex of proteins, filaments, and microtubules that spans two membranes with elements on both flagellum and cell body sides. How FAZ components are carried into place to form this complex is poorly understood. Here, we show that the trypanosome-specific kinesin KIN-E is required for building the FAZ in bloodstream-form parasites. KIN-E is localized along the flagellum with a concentration at its distal tip. Depletion of KIN-E by RNAi rapidly inhibits flagellum attachment and leads to cell death. A detailed analysis reveals that KIN-E depletion phenotypes include failure in cytokinesis completion, kinetoplast DNA missegregation, and transport vesicle accumulation. Together with previously published results in procyclic form parasites, these data suggest KIN-E plays a critical role in FAZ assembly in *T. brucei*.

## Abstract (executive summary)

The flagellum of *Trypanosoma brucei* is anchored to the cell body *via* a multiprotein structure termed the flagellum attachment zone (FAZ). How FAZ components are carried into place is poorly understood.

The trypanosome-specific kinesin KIN-E is required for FAZ assembly, flagellar attachment, and cell viability in bloodstream-form parasites.

The essential function of KIN-E in bloodstream form *T. brucei* raises the possibility that KIN-E-targeting drugs could be developed to treat human and animal African trypanosomiasis.

## INTRODUCTION

*Trypanosoma brucei* ssp. are unicellular flagellated parasites endemic to Sub-Saharan Africa that cause human African trypanosomiasis (also known as sleeping sickness; Franco *et al.*, 2020) and *nagana* disease in cattle (Giordani *et al.*, 2016). These parasites are transmitted by the bite of infected tsetse flies, proliferate extracellularly in human blood, and eventually reach the central nervous system (Brun *et al.*, 2010). *T. brucei* escapes the host adaptive immune system by rapidly replacing its entire surface coat of variable surface glycoprotein (VSG) and by removing bound antibodies (Overath *et al.*, 1997; Engstler *et al.*, 2004, 2007).

To adapt to the different environments encountered during its complex life cycle, *T. brucei* undergoes major cytoskeletal rearrangements as it transitions from procyclic trypomastigote (procyclic form or PCF) in the tsetse fly gut, to epimastigote in the salivary glands, and back to the bloodstream trypomastigote (bloodstream form or BSF) within the bloodstream of the mammalian host (Matthews, 2005). The parasite relies primarily on its microtubule cytoskeleton, which is composed of sub-pellicular microtubules, the microtubule quartet (MtQ), and the flagellar axoneme consisting of nine microtubule doublets surrounding a central pair of microtubules. In particular, the flagellum functions in movement, coordination of cytokinesis, and as a sensory organelle (Langousis and Hill, 2014). The *T. brucei* axoneme, together with the associated paraflagellar rod (PFR; Vaughan, 2010), are contained within the flagellar membrane. The flagellum originates inside the cell body from the basal body (BB), which physically connects it to the mitochondrial DNA (kinetoplast DNA or kDNA) via the tripartite attachment complex (TAC; Robinson and Gull, 1991; Ogbadoyi *et al.*, 2003). The flagellum then exits the cell from a membrane invagination called flagellar pocket (FP), which is depleted of sub-pellicular microtubules, enabling endocytosis and exocytosis take place (Allen *et al.*, 2003; Field and Carrington, 2009). At the flagellum exit point, a cytoskeletal structure called the flagellar pocket collar (FPC) tightens the FP around the flagellar membrane (Bonhivers *et al.*, 2008; Perdomo *et al.*, 2016). During the cell division cycle, a new flagellum is assembled from the BB and, as it grows, its distal tip is tethered to the old flagellum. In PCF cells, the tethering structure is called the flagellar connector (Moreira-Leite *et al.*, 2001; Briggs *et al.*, 2004; Davidge *et al.*, 2006), whereas in BSF cells it is called the flagellar groove (Hughes *et al.*, 2013; Smithson *et al.*, 2022). Cytokinesis proceeds by dividing cells between the new and old flagella, and thus each daughter cells inherits one flagellum.

Unlike for many ciliated cells, the flagella of *T. brucei* connect to the cell body along much of its length via the flagellum attachment zone (FAZ), a multiprotein complex of microtubules, filaments, and other proteins that spans and anchors the flagellar and cell body membranes (Gull, 1999). Several FAZ components play essential roles in regulating cell length, organelle positioning, and cell division (Zhou *et al.*, 2014; Sunter and Gull, 2016). On the cell body side, the FAZ includes the FAZ filament (Zhou *et al.*, 2015) and the MtQ. Transmembrane proteins including FLA1 and FLA1BP connect the cell body and flagellar membranes (Nozaki *et al.*, 1996; Sun *et al.*, 2013). On the flagellar side, FAZ connectors such as FLAM3 link the axoneme to the adhesion region (Ralston *et al.*, 2009). Because of the functions of the flagellum and FAZ, downregulating components can have dramatic effects on cell physiology. For example, in PCF cells, downregulation of FLAM3 drastically reduces FAZ length and leads to a transition into the epimastigote form (Rotureau *et al.*, 2014; Sunter *et al.*, 2015). In BSF, FLAM3 is localized in flagella with a concentration at the distal tip of the new flagellum in the vicinity of the flagellar groove (Smithson *et al.*, 2022), and its depletion leads to flagellar detachment and a severe cytokinesis defect, highlighting differences in functions depending on the life cycle stage (Sunter *et al.*, 2015).

In all eukaryotes, the assembly of the flagellar axoneme and other functions of the microtubule cytoskeleton require the activity of kinesin motor proteins, which transport cargoes such as protein complexes, organelles, and chromosomes along microtubules, and regulate microtubule dynamics (Hirokawa and Noda, 2008). The genome of *T. brucei* encodes ∼ 50 kinesin-like proteins, many of which are divergent at the level of primary amino acid sequence (Berriman *et al.*, 2005). Some of these kinesins are classified in families that are unique to kinetoplastids, and some so-called orphan-kinesins cannot be classified within any known kinesin subfamily from other species (Wickstead and Gull, 2006; Wickstead *et al.*, 2010). The orphan and kinetoplastid-specific kinesin KIN-E (Tb927.5.2410; Aslett *et al.*, 2010) was shown in PCF parasites to play an essential role in maintaining trypomastigote morphology and targeting the FAZ component FLAM3 to the flagellum for FAZ assembly (An and Li, 2018). Although the importance of stage-specific studies of flagellar protein function has recently been emphasized (Bachmaier *et al.*, 2022), the role of KIN-E in the medically relevant BSF cells has remained undetermined.

In this work, we characterize the localization and function of KIN-E in BSF parasites. We show an essential role for KIN-E in cell survival and FAZ assembly. We also describe morphological phenotypes induced by KIN-E depletion, including impaired cytokinesis and kinetoplast DNA (kDNA) segregation. Moreover, we find KIN-E is important for FLAM3 localization, suggesting FLAM3 is a cargo of KIN-E in BSF parasites.

## RESULTS

### KIN-E localizes along the flagellum with a concentration at the flagellar tip

To examine the function of KIN-E in BSF parasites, we first sought to localize the KIN-E protein. We raised a polyclonal antibody in rabbits that recognizes the C-terminus of the protein (aa 1105–1339; Supplemental Figure S1A). By Western blotting, the antibody detected the recombinant protein expressed in bacteria (Supplemental Figure S1, B–D; tagged with glutathione-*S*-transferase [GST]) and recognized a single band of ∼ 150 kDa in *T. brucei* cell extract, corresponding to the annotated molecular mass of 149,644 Da (Supplemental Figure S1E).

**FIGURE 1:**
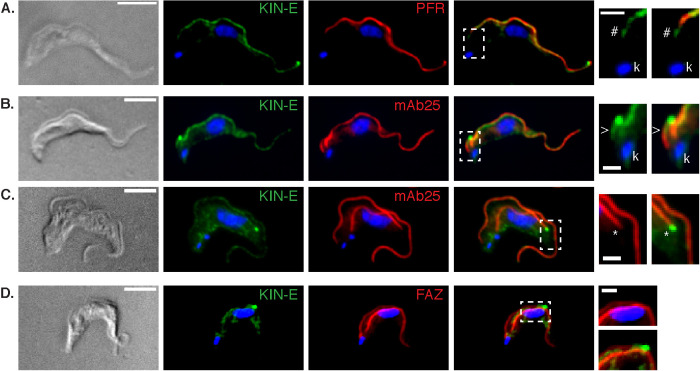
KIN-E localizes along the flagellum with a concentration at the flagellar tip. (A–D) *T. brucei* BSF 90-13 cells visualized by differential interference contrast (DIC) microscopy, or immunofluorescence microscopy using anti-KIN-E (green) and also stained for DNA (blue, DAPI) and the following markers (red): (A) L8C4, a PFR marker; (B–C) mAb25, an axonemal marker; and (D) L3B2, a FAZ marker. White dashed boxes correspond to insets on right that show magnified views of: (A and B) the proximal end of the flagellum, with (A) # indicating a comparison of the proximal end of the KIN-E signal with the L8C4; and (B) > indicating a comparison of the proximal end of the KIN-E signal with mAb25; (C) KIN-E staining at the new flagellum tip (*) compared with mAb25; (D) Brightness enhanced image of KIN-E on a new flagellum adjacent to the FAZ signal. KIN-E staining can also be seen at the distal flagellar tip in (A) and on the distal tip of very short (B) or already longer new flagella (C and D). (k) indicates the kDNA. Scale bars 5 µm (left) and 1.5 µm (right).

By immunofluorescence microscopy in BSF cells, the anti-KIN-E antibody stained along the old and new flagella with a signal enrichment at the tip of the new flagellum (Figure 1). Occasional signal enrichment could also be seen on the tip of the old flagellum, and nonspecific cytoplasmic staining was also sometimes observed. KIN-E signal was seen at the distal tips of both short (Figure 1B) and long (Figure 1C) new flagella, suggesting it selectively tracks the growing flagellum tip in the flagellar groove (Hughes *et al.*, 2013; Smithson *et al.*, 2022).

To gain more detailed insight into KIN-E localization at the proximal end of the flagellum and along its length, we compared its distribution with that of known flagellar markers. In cells stained for KIN-E and a marker of the PFR (L8C4; Kohl *et al.*, 1999), KIN-E extended more towards the proximal end of the flagellum (nearer to the BB) than the PFR (Figure 1A). On the other hand, in cells stained for KIN-E and the axonemal marker mAb25 (Dacheux *et al.*, 2012), the mAb25 signal extended more towards the proximal end of the flagellum than the KIN-E signal (Figure 1B). We also stained for KIN-E and a marker of the cell-body side of the FAZ (L3B2; Kohl *et al.*, 1999), and found that KIN-E and the FAZ followed parallel paths, but did not overlap (Figure 1D), indicating KIN-E is found on the flagellum side of the FAZ. Together, these results indicate that, in addition to the flagellar tip localization, KIN-E staining initiates at the proximal end of the flagella distal to the start of the axoneme but proximal to the start of the PFR, likely corresponding to a region near the FPC.

### KIN-E is essential for bloodstream-form *T. brucei* survival

**FIGURE 2:**
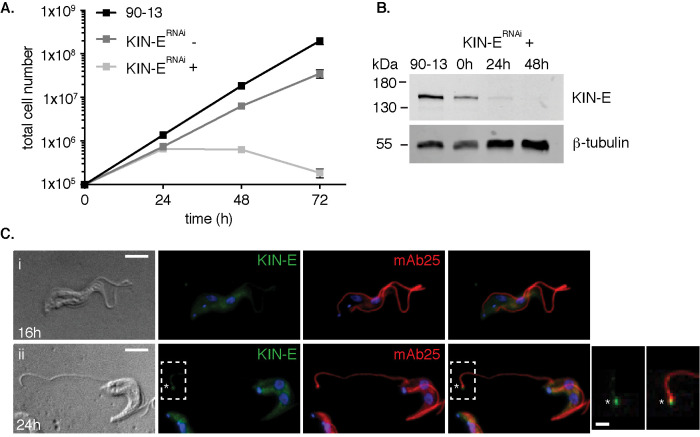
KIN-E is essential for bloodstream-form *T. brucei* survival. (A) Growth curve of the parental cell line (90-13) compared with uninduced (-) and induced (+) KIN-E^RNAi^ cells. Error bars show SE, *n* = 3 independent experiments. (B) KIN-E protein expression during RNAi induction analyzed by Western blotting with anti-KIN-E antibody and anti-β-tubulin antibody as a loading control. Equal cell numbers (5 × 10^6^ cells) were loaded per lane. (C) KIN-E^RNAi^ cells visualized by DIC or immunofluorescence microscopy at (i) 16 hpi or (ii) 24 hpi using anti-KIN-E (green) and mAb25 (red) as an axonemal marker. Residual new flagellar tip signal in (ii) is marked with an asterisk (*). Scale bars 5 µm (left) and 1.25 µm (right).

To study the function of KIN-E in BSF parasites, we generated a stable and tetracycline-inducible RNAi cell line (KIN-E^RNAi^). We monitored cell growth of the parental cell line (90-13), and of uninduced and induced KIN-E^RNAi^ cells. Upon KIN-E RNAi induction, we observed growth arrest starting at 24-h postinduction (hpi), followed by cell death (as measured by a decrease in cell number) within 72 hpi (Figure 2A). The efficiency of KIN-E knockdown was confirmed by Western blotting. At 24 hpi, KIN-E expression dropped below 20% of that in uninduced cells, and at 48 hpi KIN-E was not detected (Figure 2B). KIN-E signal was also monitored by immunofluorescence microscopy at 16 hpi (Figure 2Ci) and 24 hpi (Figure 2Cii) in cells counterstained for the flagellar axoneme (mAb25). KIN-E signal was diminished along the flagellum after 16 and 24 h (although some residual signal remained at flagellar tip; compare with Figure 1). These data indicate that KIN-E expression is efficiently downregulated by RNAi, resulting in premature cell death. Thus, KIN-E is essential for BSF parasite survival.

### KIN-E is required for the attachment of the newly synthetized flagellum, but not for flagellum biogenesis or beating

**FIGURE 3:**
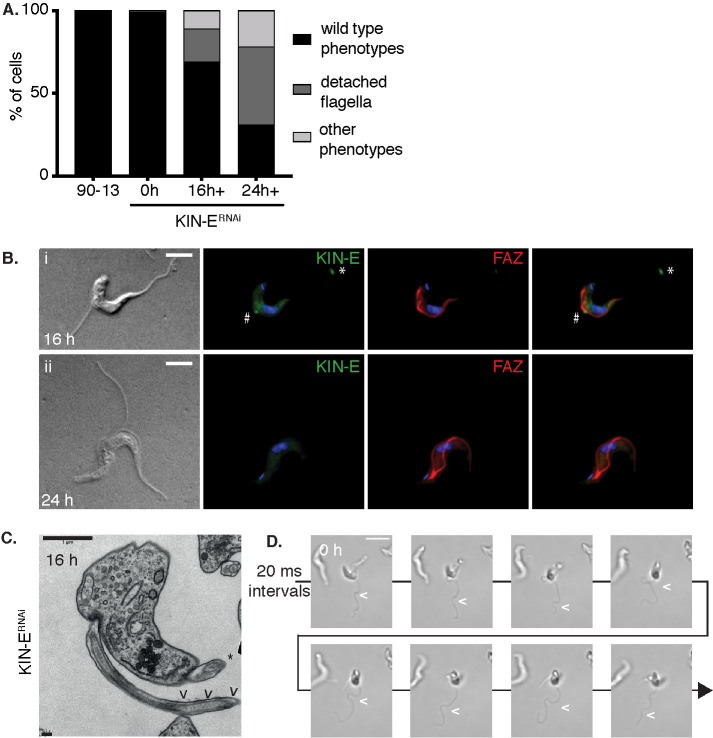
KIN-E is required for the attachment of the newly synthetized flagellum. (A) Graph of the percentage of KIN-E^RNAi^-uninduced or -induced cells with detached flagella or other abnormal phenotypes at 16 and 24 hpi compared with parental cell line 90-13 >200 cells per condition, *n* = 3 independent experiments. (B) KIN-E^RNAi^ cells visualized by DIC or immunofluorescence microscopy at (i) 16 hpi or (ii) 24 hpi, using anti-KIN-E (green) and L3B2 (red) as a FAZ marker. Residual KIN-E signal is visible at the point of detachment of a new flagellum (#) and at the tip of old flagellum (*). (C) TEM image of KIN-E^RNAi^ cells at 16 hpi. Arrowheads (<) indicate the detached new flagellum. The attached old flagellum on the same cell is marked with an asterisk (*). Upper left scale bar 1 μm; lower left scale bar 0.2 μm. (D) Video frames of KIN-E^RNAi^ cells at 24 hpi, acquired every 20 ms. Flagellar wave in a detached flagellum is indicated with an arrowhead. Scale bar 10 µm. Also see Supplemental Video S3.

In observing the morphology of KIN-E^RNAi^ cells during the first 24 hpi, we noted that many had partially detached new flagella, although cells otherwise looked relatively normal (Figures 2C and 3A). We quantified the percentage of cells with a detached flagellum and found that it increased from ∼20% at 16 hpi to ∼50% at 24 hpi (Figure 3A). We hypothesized that failure in flagellum attachment was caused by improper FAZ assembly. To assess FAZ integrity, we stained KIN-E^RNAi^ cells for a FAZ marker (L3B2) at 16 hpi and 24 hpi. We observed that FAZ staining was interrupted at the location where the new flagellum detached from the cell body, although the FAZ staining associated with the old flagellum was unaffected (Figure 3, Bi and Bii). This suggests that new flagella become detached, whereas old flagella remain attached. Detached new flagella and attached old flagella were also visible by transmission electron microscopy (TEM) at 16 hpi (Figure 3C). These findings suggest that KIN-E plays a role in FAZ assembly, and that downregulating KIN-E function causes defects in this process that result in detachment of the new flagellum.

We also investigated whether motility of the detached new flagellum was affected by KIN-E depletion. We recorded movies of 90-13 parental cells as well as KIN-E^RNAi^ cells with detached flagella at 24 hpi (Figure 3D; Supplemental Videos S1–S4). We observed active beating of detached as well as attached flagella (Supplemental Video S4 features an unusual abnormal dividing cell with two attached and two detached beating flagella). Altogether, this suggests that KIN-E is essential for flagellar attachment, but not for flagellar biogenesis or beating.

**Figure:** Movie S1 Flagellum beating in a 90‐13 cell. Frames acquired every 20 ms, plays at 25 frames/sec.

**Figure:** Movie S2 Flagella beating in a dividing 90‐13 cell. Frames acquired every 20 ms, plays at 25 frames/sec.

**Figure:** Movie S3 Detached flagellum beating in a KIN‐E^RNAi^ cell at 24 hpi. Frames acquired every 20 ms, plays at 25 frames/sec.

**Figure:** Movie S4 Detached flagella beating in a dividing KIN‐E^RNAi^ cell at 24 hpi. Frames acquired every 20 ms, plays at 25 frames/sec.

#### KIN-E depletion disrupts cell division.

**FIGURE 4:**
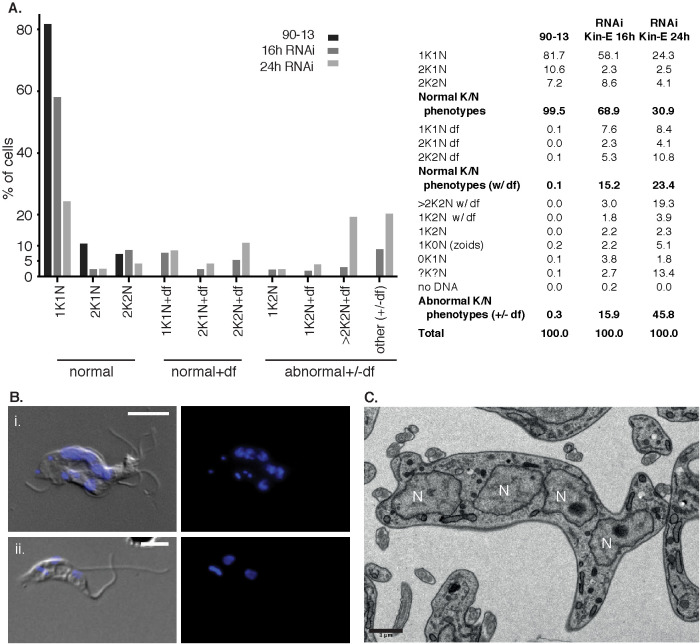
KIN-E depletion causes a failure in cytokinesis. (A) Graphical and tabular representations of phenotypic counts with a focus on DNA content in KIN-E^RNAi^ cells at 16 and 24 hpi compared with the parental cell line 90-13. The categories were defined as follows: normal kinetoplast (K) and nucleus (N) phenotypes (1K1N, 2K1N, and 2K2N); normal K/N phenotypes with detached flagella (+df); abnormal K/N phenotypes with or without detached flagella (+/-df), including cells with a single kDNA but two nuclei (1K2N), multinucleated cells (>2K2N), and other phenotypes (anucleated cells (1K0N, zoids); cells without a kinetoplast (0K1N); cells with indeterminate DNA content (?K?N); and cells without detectable DNA (no DNA)). *n* > 200 cells per cell line. (B) Examples of (i) a multinucleated cell (>2K2N) and (ii) a 1K2N cell. Nuclei and kinetoplasts were stained with DAPI (blue); cell bodies and flagella were visualized with DIC. Scale bars 5 μm. (C) Thin section TEM micrograph of KIN-E^RNAi^ population induced for 24 hpi, showing a cell with multiple nuclei (N). Scale bar 1 μm.

Previous studies reported that defects in FAZ assembly often lead to secondary defects in cytokinesis (Robinson *et al.*, 1995; Kohl *et al.*, 2003). To determine the effect of KIN-E depletion on cell cycle progression and cytokinesis in the BSF, we performed a comprehensive phenotypic analysis, focusing on quantifying nuclear DNA (N) and kinetoplast DNA (kDNA or K) content (Figure 4A). We divided the cell population in three categories: cells with wild-type DNA content (1K1N, 2K1N, and 2K2N) and attached flagella; cells with wild-type DNA content and detached flagella; and cells with abnormal DNA content, with or without detached flagella. The latter category included: multinucleated cells, indicating failed cytokinesis (>2K2N; Figure 4, A and Bi); cells with one kDNA but two nuclei, indicating failed kDNA replication/division (1K2N; Figure 4Bii); and other phenotypes (1K0N, 0K1N, and indeterminate DNA content [?K?N]). As expected, almost all 90-13 parental cells had wild-type DNA content and attached flagella (Figure 4A). In contrast, KIN-E^RNAi^ cells at 16 hpi and 24 hpi showed an increasing percentage of cells with abnormal DNA content, including 20% multinucleated cells at 24 hpi (Figure 4A). Multinucleated cells were also observed by TEM (Figure 4C). Thus, KIN-E depletion leads to defects in cytokinesis, likely as a consequence of disturbed FAZ assembly.

#### KIN-E depletion impacts kDNA segregation.

**FIGURE 5:**
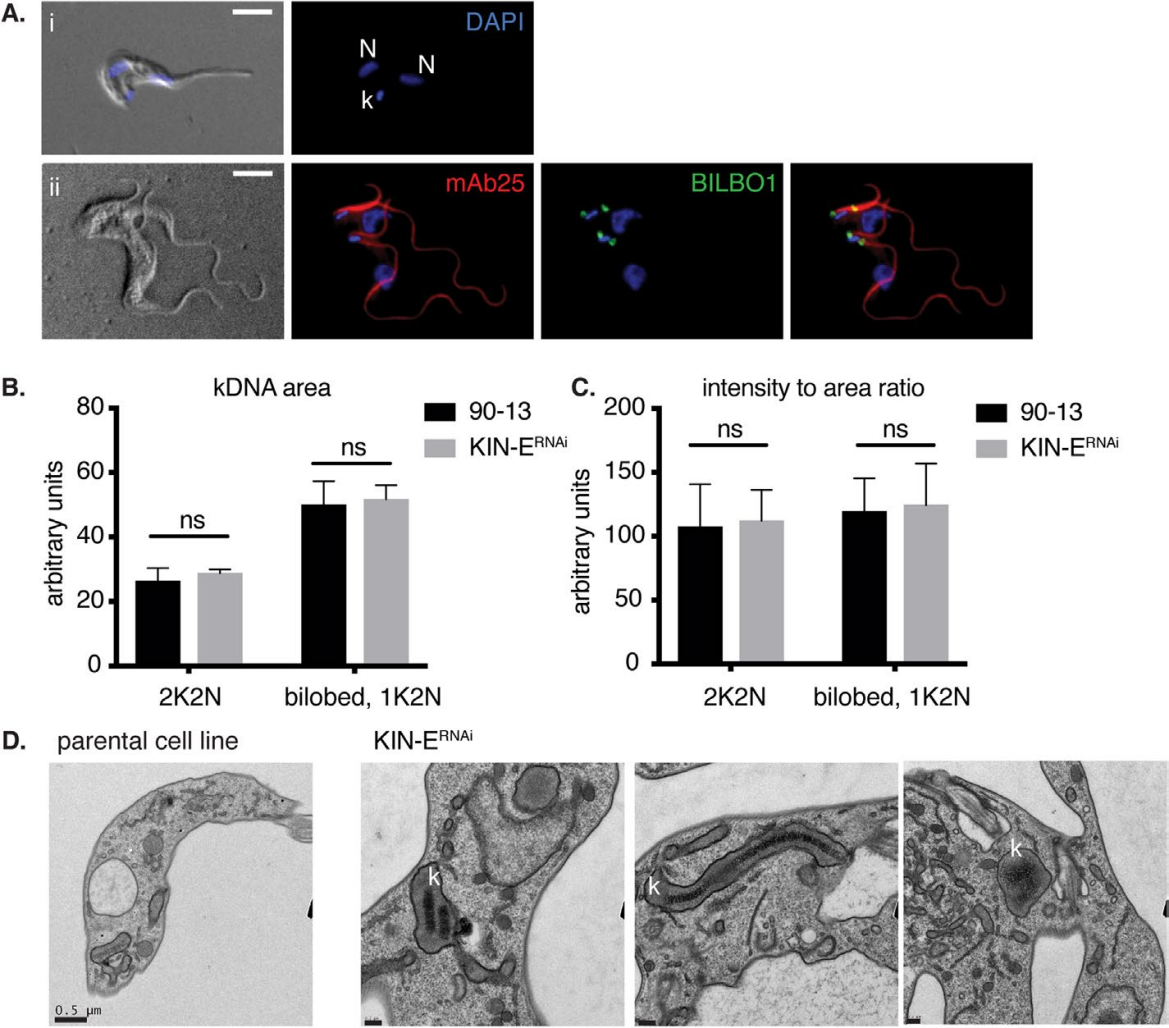
KIN-E influences kDNA segregation. (A) KIN-E^RNAi^ cells at 24 hpi, showing (i) a 1K2N cell with a single enlarged kDNA and two nuclei, and (ii) a 2K2N cell with four flagella stained with mAb25 antibody (red), and four FPCs stained with anti-BILBO1 antibody (green). Scale bars 5 μm. (B) kDNA area and (C) ratio of kDNA signal intensity/area comparing single kDNAs in KIN-E^RNAi^ induced 2K2N cells versus parental 90-13 2K2N cells, as well as the single kDNA (1K2N) in KIN-E^RNAi^-induced cells versus the bilobed-shaped kDNA in parental 90-13 cells. Quantification of 100 cells per condition, *n* = 3 independent experiments. Statistical comparisons between strains were performed using a *t* test, ns = nonsignificant. (D) Thin section TEM micrographs of parental cells and KIN-E^RNAi^ cells at 16, 16, and 24 hpi, showing different abnormal kDNA (k) configurations. Scale bars 0.5 μm for the parental cell line, and 0.2 μm for KIN-E^RNAi^.

As mentioned above, we observed that 1K2N cells represented up to 7% of KIN-E^RNAi^ cells at 16 and 24 hpi (Figures 4A and 5Ai). We sought to further test whether these were defective in kDNA duplication or kDNA segregation. Because the kDNA is physically attached to the flagellum (Robinson and Gull, 1991), we first imaged flagella (by staining the axoneme with mAb25) and FPC structures (by staining for BILBO1; Esson *et al.*, 2012) in 1K2N cells (Figure 5Aii). We found that each kDNA was attached to two separated FPCs and two flagella, suggesting that the kDNA had duplicated but not segregated.

To further test for kDNA duplication/segregation, we separately measured the area of each single DAPI-stained kDNA in parental 90-13 and induced KIN-E^RNAi^ 2K2N cells, as well as each kDNA in induced KIN-E^RNAi^ 1K2N cells. The mean area of kDNA in KIN-E^RNAi^ 1K2N cells was twice as large as each single kDNA in 2K2N cells (Figure 5B). Additionally, the area of kDNA in 1K2N KIN-E^RNAi^ was comparable to the dividing bilobed-kDNA (Gluenz *et al.*, 2011) in the parental cell line (Figure 5B). We also measured kDNA signal intensities and calculated the intensity:area ratio. On average, the kDNAs in 1K2N KIN-E^RNAi^ and dividing bilobed-kDNA parental cells were both twice as intense and twice as large as the single kDNAs in 2K2N cells, resulting in a comparable intensity:area ratio (Figure 5C). Finally, by TEM we observed in seven of 72 sections that the kDNA was in an atypical configuration, appearing as two compacted or one elongated disk, or as a disorganized structure (Figure 5D). From these data, we conclude KIN-E depletion does not impact mitochondrial DNA duplication but does impact mitochondrial DNA segregation.

#### KIN-E is important for vesicular trafficking near the FP.

**FIGURE 6:**
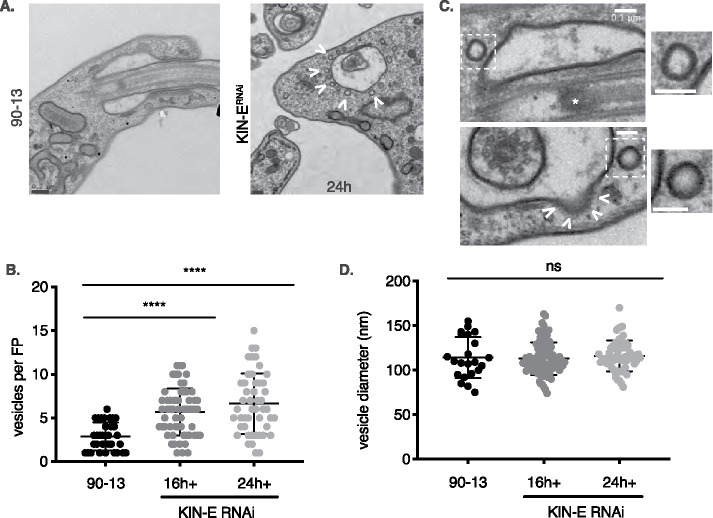
KIN-E downregulation leads to vesicle accumulation near the FP. (A) Thin section TEM micrographs comparing the FP region of the parental cell line 90-13 with that of a KIN-E^RNAi^ at 24 hpi. Scale bar 0.2 μm. (B) Number of vesicles per FP for the parental cell line 90-13 and KIN-E^RNAi^ cells at 16 and 24 hpi. *n* > 200 vesicles per condition. Bars show group mean as well as top and bottom quartiles. Pairwise statistical comparisons were performed using a *t* test, **** *p* < 0.0001. (C) Thin section TEM micrographs show vesicles near the FP in KIN-E^RNAi^ cells. White dashed boxes show magnified views of individual vesicles. The upper panel shows a longitudinal cross section of the flagellum showing the basal plate (*). The lower panel shows a transverse cut around the basal plate of the flagellum. Arrowheads mark a clathrin-coated vesicle budding from the FP. Scale bar 0.1 μm. (D) Vesicle diameter was measured from TEM micrographs using ImageJ. *n* = 165 total vesicles measured. Pairwise statistical comparisons were performed using a *t* test, ns = nonsignificant.

In the vicinity of the FP in TEM sections, we also observed an increased number of vesicles in the KIN-E^RNAi^-induced cells compared with parental cells (Figure 6A). At 16 hpi, KIN-E^RNAi^ cells had an average of 6 ± 0.4 vesicles, and at 24 hpi, KIN-E^RNAi^ cells had an average of 7 ± 0.5 vesicles, whereas parental 90-13 cells had 3 ± 0.5 vesicles per FP (Figure 6B). The diameter of vesicles was indistinguishable in parental versus induced KIN-E^RNAi^ cells, and averaged 115 ± 2 nm (Figure 6, C and D). In BSF *T. brucei*, endocytosis occurs at the FP via large clathrin-coated vesicles (135 nm in diameter) containing VSG (Grünfelder *et al.*, 2003; Engstler *et al.*, 2005). We observed clathrin-coated vesicles in the process of being internalized (Figure 6C). The shape and size of the observed vesicles in the parental cell line, uninduced KIN-E^RNAi^ cells, and induced KIN-E^RNAi^ population were similar to endocytic vesicles after shedding their clathrin coat. The luminal side of the vesicles showed an electron-dense material of the same thickness as the coat on the cell surface and flagellar membrane (Figure 6C), suggesting this material is VSG. For these reasons, we speculate that these vesicles near the FP are endocytic vesicles.

#### KIN-E is required for FLAM3 localization to the new flagellar tip.

**FIGURE 7:**
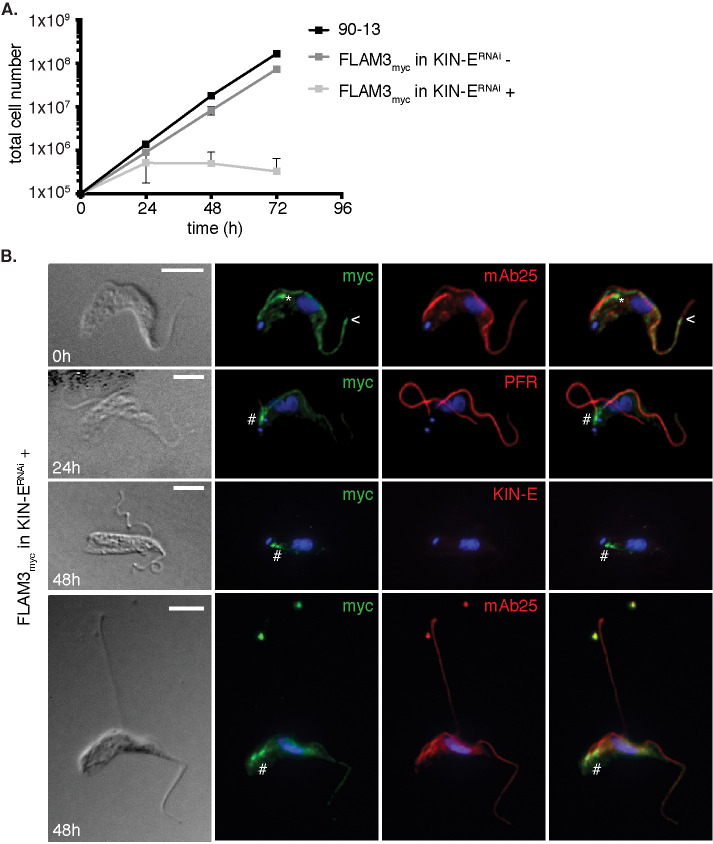
FLAM3 is a KIN-E cargo. (A) Growth curve of the parental cell line 90-13 compared with FLAM3_myc_ in KIN-E^RNAi^- uninduced (-) and -induced (+) cells. (B) FLAM3_myc_ KIN-E^RNAi^ cells visualized by DIC or immunofluorescence microscopy. Immunofluorescence analysis of endogenously expressed FLAM3-10_myc_ in KIN-E^RNAi^ uninduced (0 hpi) and induced cells at 24 and 48 hpi using anti-myc to detect FLAM3 (green), and mAb25 or PFR (red) as a flagellar marker. Asterisk (*) marks the bright FLAM3 signal at the new flagellar tip, arrowheads (<) show the point where FLAM3 signal stops near the distal end of the flagellum, hash tag (#) indicates FLAM3 signal accumulation at the proximal end of the flagellum. The two bright dots near the top of the bottom row 48 hpi image are likely flagellar debris from dead cells. Scale bar 5 μm.

Our observation that KIN-E depletion disrupts FAZ formation suggests that it functions to transport cargoes that are known components of the FAZ (LaCount *et al.*, 2002; Kohl *et al.*, 2003; Vaughan *et al.*, 2008; Rotureau *et al.*, 2014; Sunter *et al.*, 2015; Moreira *et al.*, 2017; An *et al.*, 2020). One potential KIN-E cargo is FLAM3, which was previously identified as a FAZ component that is located on the flagellar side, accumulates at the new flagellar tip, and depending on the cell cycle stage, shows weak or no localization to the old flagellar tip (Rotureau *et al.*, 2014; Sunter *et al.*, 2015; Smithson *et al.*, 2022). Therefore, we tested whether KIN-E is important for FLAM3 localization in BSF cells. We endogenously tagged FLAM3 at its C-terminus with a 10x myc tag (FLAM3_myc_) within the KIN-E^RNAi^ cell line background. The growth of uninduced cells was unaffected by the expression of FLAM3_myc_ (Figure 7A). We localized FLAM3_myc_ within the old and the new flagellum by immunofluorescence microscopy and observed signal enrichment at the new flagellar tip (Figure 7B), but no signal at the old flagellar tip (Figure 7B). We next tested whether KIN-E depletion had an effect on FLAM3 localization by examining the distribution of FLAM3 in induced KIN-E^RNAi^ cells at 24 and 48 hpi (Figure 7B). As described above for KIN-E^RNAi^ cells (Figure 2A), induced KIN-E^RNAi^ FLAM3_myc_ cells died within 72 hpi and showed detached new flagella. FLAM3 was at the tip of new flagella at 0 hpi. However, at 24 and 48 hpi, FLAM3 was confined to the proximal end of new flagella (Figure 7B), between the kDNA and the origin of the PFR. The old flagella still showed weak FLAM3 staining (labeled with mAb25) at 24 hpi, whereas flagellar FLAM3 signal was nearly absent at 48 hpi. As control, we used KIN-E antibody to confirm that KIN-E was properly depleted at 48 hpi (Figure 7B), while FLAM3 was present at the proximal end of the flagellum and diffuse within the cytosol (Figure 7B). The altered FLAM3 localization suggests that in BSF parasites, KIN-E is required for FLAM3 transport into the flagellum.

## DISCUSSION

We investigated the localization and function of the kinetoplastid-specific kinesin KIN-E in BSF *T. brucei*. We found that KIN-E is localized within the flagellum, with an enrichment at the distal tip of growing new flagella in the vicinity of the flagellar groove. We further observed that KIN-E is essential for cell survival of *T. brucei* BSF parasites. KIN-E is necessary for attachment of the newly synthesized flagellum and biogenesis of the FAZ. In the absence of KIN-E expression, cytokinesis fails, as does kDNA segregation in a subset of cells. Our work establishes an essential role for KIN-E in FAZ assembly and cell viability in BSF trypanosomes.

Our phenotypic analysis in BSF confirms and extends the characterization of KIN-E function in PCF trypanosomes (An and Li, 2018). In PCF cells, KIN-E is also localized to the flagellum and enriched at the flagellar tip (Dean *et al.*, 2017; An and Li, 2018). However, in BSF cells, we observe significant KIN-E signal enhancement at the tips of new flagella, whereas in PCF cells it is seen at the tips of both old and new flagella (Dean *et al.*, 2017; An and Li, 2018). Furthermore, in PCF cells, KIN-E is important for normal growth rate (An and Li, 2018), although we find it is essential for viability in BSF cells. KIN-E depletion in PCF cells also induces the repositioning of the kDNA and the production of epimastigote-like cells (An and Li, 2018), outcomes we do not observe in BSF cells.

A key function of KIN-E in both PCF and BSF cells is in flagellar attachment. KIN-E depletion in PCF cells causes a failure in attachment of newly synthetized flagella (An and Li, 2018), similar to what we observe in BSF cells. In both PCF and BSF parasites, cells with detached new flagella contain a full-length old FAZ filament but a short new FAZ filament, suggesting premature termination of FAZ synthesis. The old FAZ filament is not affected by KIN-E depletion, suggesting that once this component of the FAZ on the cell body side is synthesized, KIN-E is no longer required for its maintenance. However, on the flagellar side, FAZ maintenance could be affected by KIN-E depletion. Furthermore, KIN-E is not required for flagellar length, which *in T. brucei* is controlled by the intraflagellar transport (IFT) machinery (Kohl *et al.*, 2003; Absalon *et al.*, 2008), as it is in other organisms (Taschner and Lorentzen, 2016). In further support of the notion that KIN-E is not required for flagellar function, we found that detached flagella continue to beat in KIN-E-depleted BSF cells.

One cargo of KIN-E in PCF cells is FLAM3 (An and Li, 2018), a component of the FAZ on the flagellar side (Rotureau *et al.*, 2014). Upon KIN-E depletion in PCF cells, FLAM3 becomes diffusely localized within the cytosol (An and Li, 2018). Our work suggests that FLAM3 is also a cargo of KIN-E in BSF cells. Interestingly, upon KIN-E depletion in BSF, we find that FLAM3 accumulates at the proximal end of the flagellum in an area that may overlap with the transition zone, which has been described as the “gate” that controls transport of components into the flagellum (Reiter *et al.*, 2012; Dean *et al.*, 2016). There may also be other KIN-E cargoes, for example, the proteins ClpGM6 and FAZ27, which colocalize and interact with FLAM3 on the flagellar side of the FAZ (Sunter *et al.*, 2015; An *et al.*, 2020). Future research will establish their interactions and the mechanism of FAZ assembly.

We observed a second major phenotype upon KIN-E depletion in BSF cells, which is the accumulation of multinucleated and multiflagellated cells, as well as a small population of zoids, indicative of failure in cell division or aberrant cell division. Normal flagellum structure and function is important for cell division in *T. brucei*, and in BSF *T. brucei,* flagellar defects cause a failure in cell division and cell inviability (Broadhead *et al.*, 2006; Ralston *et al.*, 2006; Hammarton *et al.*, 2007; Li and Wang, 2008; Ikeda and de Graffenried, 2012). RNAi knockdown of IFT proteins also produces defects in flagellum construction and causes impaired cytokinesis (Kohl *et al.*, 2003; Bertiaux *et al.*, 2018; Douglas *et al.*, 2020). *T. brucei* cytokinesis also requires flagellar attachment, and loss of FAZ proteins like FAZ1 (Vaughan *et al.*, 2008), FLA1 (LaCount *et al.*, 2002), FAZ10 (Moreira *et al.*, 2017), or FLAM3 (Rotureau *et al.*, 2014; Sunter *et al.*, 2015) leads to defects in cytokinesis. Thus, cytokinesis failure and cell inviability in KIN-E-depleted BSF cells is likely a secondary consequence of defects in FAZ synthesis and flagellar attachment.

We found that depletion of KIN-E also causes other phenotypes. For example, a subset of KIN-E-depleted cells (lt 10%) fail in segregating their kDNA. In *T. brucei*, kDNA is physically linked to the flagellar BBs *via* the TAC complex (Robinson and Gull, 1991; Ogbadoyi *et al.*, 2003), and its segregation is orchestrated by the movements of the BBs (Lacomble *et al.*, 2010). However, the forces involved in this process remain mostly unknown. It has been suggested that the MtQ could drive BB movement and consequently kDNA segregation (Lacomble *et al.*, 2010). Moreover, a properly formed FAZ is indispensable for BB segregation and cell division (Kohl *et al.*, 2003). Failure in kDNA segregation may therefore be a secondary consequence of impaired FAZ formation. A separate phenotype observed upon KIN-E depletion is the accumulation of vesicles around the FP that contain VSG in their lumen. Vesicle accumulation is not accompanied by an expansion of the FP, as occurs following depletion of proteins important for protein entry into or endocytosis within the FP (Allen *et al.*, 2003; García-Salcedo *et al.*, 2004; Morriswood and Schmidt, 2015), suggesting that there is no global inhibition of endocytosis. The accumulated vesicles may be endocytic vesicles that have shed their clathrin coat, or exocytic vesicles that contain cell-body side FAZ components that could not be assembled due to KIN-E depletion. Further investigations could elucidate how KIN-E impacts kinetoplast segregation and vesicular trafficking in the vicinity of BBs and FP.

The further study of KIN-E and its cargos could help elucidate how the FAZ is assembled and maintained in *T. brucei*. Moreover, KIN-E is a kinetoplastid-specific kinesin with orthologs in the related organisms *Trypanosoma cruzi* and *Leishmania spp.* (Aslett *et al.*, 2010). The fact that KIN-E is essential for parasite survival makes it a potential drug target. Kinesin inhibitors have been identified with promising drug-like properties and have been tested as anti-cancer drugs (Mayer *et al.*, 1999; Khathi *et al.*, 2018; Wu *et al.*, 2018). This raises the possibility that KIN-E-targeting drugs could be developed to treat human and animal African trypanosomiasis, as well as Chagas disease and Leishmaniasis.

## MATERIALS AND METHODS

Request a protocol through *Bio-protocol*.

### Cell lines, growth conditions, and transfections

*T. brucei* strain 427 90-13 BSF cells (Wirtz *et al.*, 1999) were cultured at 37°C in HMI-9 medium (Hirumi and Hirumi, 1989) supplemented with 10% FBS (Atlanta Biologicals), 2.5-μg/ml G418 (InvivoGen), and 5-μg/ml hygromycin (InvivoGen).

Plasmid transfections into 90-13 cells were performed using the Amaxa Nucleofector system (Lonza) with program X-001 as described previously (Burkard *et al.*, 2007), and with Tb-BSF buffer (90-mM Na_2_HPO_4_, 5-mM KCl, 0.15-mM CaCl_2_, 50-mM HEPES, pH 7.3; Schumann Burkard *et al.*, 2011). Stable cell lines were selected by culturing cells in medium containing 2.5-μg/ml phleomycin (InvivoGen) and/or 10-μg/ml blasticidin (InvivoGen). Expression of double-stranded RNA was induced by adding 1-μg/ml tetracycline (Sigma-Aldrich) to the culture medium.

### Plasmid construction

For RNAi silencing of KIN-E expression in *T. brucei*, we PCR-amplified a DNA segment (bp 1798–2333) of the KIN-E gene (Tb927.5.2410) from genomic DNA isolated from *T. brucei* 90-13 cells using the Qiagen DNeasy Blood and Tissue kit. Suitability of this segment for RNAi was confirmed using the online tool RNAit (Redmond *et al.*, 2003). The purified PCR product was inserted by standard ligation into XhoI and HindIII sites within the pZJM vector (Wang *et al.*, 2000) containing the phleomycin resistance (*ble*) gene, between two opposing T7 promoters. A total of 10 μg of the plasmid was linearized with NotI for transfection into bloodstream form *T. brucei* 90-13 cells (carried out as described above).

To generate a *T. brucei* strain expressing endogenous C-terminally myc-tagged FLAM3 (FLAM3_myc_), we used the long primer PCR transfection method described previously (Dean *et al.*, 2015). We used the pPOTv7 plasmid DNA as template for PCR amplification, which contained coding sequences for the 10× myc tag and blasticidin resistance cassette. Transfection was carried out as described above.

For protein expression and purification of GST fused to the C-terminal domain of KIN-E in *E. coli* (amino acids 1105–1339; GST-KIN-E^C-ter^), bp 3313–4017 of the corresponding gene were amplified by PCR from genomic DNA and cloned into BamHI and NotI restriction sites within the pGEX-4T-1 plasmid (GE Healthcare) such that the gene was in frame with an N-terminal GST tag (pGEX-4T-1-GST-KIN-E^C-ter^). All plasmid sequences were confirmed by DNA sequencing at the UC Berkeley DNA Sequencing Facility.

### Protein expression and purification for antibody production

The plasmid pGEX-4T-1-GST-KIN-E^C-ter^ was transformed into *E. coli* strain BL21*.* Bacteria grown in 1-l lysogeny broth (LB) with 100-µg/ml ampicillin were induced with 1-mM isopropyl-β-d-thio-galactoside (IPTG) for 2.5–4 h at 37°C. Bacteria were centrifuged (4000 × *g* for 20 min at 4°C) and resuspended in cold lysis buffer (50-mM Tris, pH 8, 50-mM NaCl, 5-mM EDTA, 0.2% Triton-X 100, 1-mM β-mercaptoethanol, 150-μM PMSF, and 1-μg/ml final volume each of leupeptin, pepstatin, and chymostatin (LPC) protease inhibitor mix). Cells were lysed on ice using a Branson Digital Sonifier 450 (at level 6 for 1 min [10-s on, 10-s off, × 3] + 30 min rest, × 3–4 cycles). Cell lysate supernatant was loaded on a Glutathione Sepharose 4B column (GE Healthcare). The column was washed with TBS (20-mM Tris, pH 7.5, 150-mM NaCl) with 5-mM EDTA, 0.1% Triton-X 100, 1-mM β-mercaptoethanol, 150-μM PMSF, and LPC. Washed bound protein was then eluted with elution buffer, 50-mM Tris pH 8.0, plus 10-mM reduced glutathione, into 10 fractions. The column fraction with the greatest protein concentration were then subjected to gel-filtration chromatography on a Superdex 75 column equilibrated with 50-mM Tris, pH 8.0, plus 150-mM NaCl. Fractions containing GST-KIN-E were collected and stored at −80˚C.

### Antibody production and purification

Two rabbits were immunized (Covance) with 1.5–2 mg of purified GST-KIN-E^C-ter^ protein, according to Covance’s 118 d protocol. KIN- E^C-ter^-specific antibodies were affinity purified as follows. Purified GST-KIN-E^C-ter^ fusion protein from pooled gel-filtration fractions was cross-linked to Affi-gel 15 beads (BioRad) in MOPS, pH 7.0, with 1-M KCl, and the beads were quenched with ethanolamine HCl, pH 8.0, at 4°C. Serum from immunized rabbits was loaded onto the column, and the column was then washed with 20-mM Tris, pH 7.6, 0.5-M NaCl and 0.2% Triton X-100. Purified bound antibody was eluted with 200-mM glycine pH 2.5, 150-mM NaCl, and 10 × 300-µl fractions were collected into fraction tubes containing 50-µl 1-M Tris-HCl, pH 8.0. To test for antibody specificity by western blotting, bacteria were cultivated at 37°C, induced for 2 h 30 min with 1-mM IPTG, harvested, boiled in sample buffer, and subjected to SDS–PAGE.

### Western blotting

Western blotting was performed using standard methods as described previously (Albisetti *et al.*, 2017), with the exception that proteins were transferred to nitrocellulose membranes (Genesee Scientific, Prometheus #84-875). Primary antibodies were used at the following dilutions: rabbit anti-KIN-E 1:2000; rabbit anti-GST 1:1000 (Welch lab); mouse anti-β-tubulin E7 1:10,000 (Developmental Studies Hybridoma Bank, University of Iowa). Secondary antibodies used were goat anti-rabbit AF790 (ThermoFisher Scientific A11367) and goat anti-mouse AF680 (ThermoFisher Scientific A21058), both diluted at 1:10,000. Images were taken using the Odyssey imaging system (Li-Cor Biosciences).

### Fluorescence microscopy

For immunofluorescence microscopy, parental and TbKIN-E^RNAi^-uninduced and -induced cells in exponential growth phase were harvested for 5 min at 1000 × g at room temperature and washed once in Voorheis’ modified PBS (vPBS; 8-mg/ml NaCl, 0.22-mg/ml KCl, 2.27-mg/ml Na_2_HPO_4_, 0.41-mg/ml KH_2_PO_4_, 15.7-mg/ml sucrose, 1.8-mg/ml glucose). The cells were resuspended in 1% paraformaldehyde (PFA) in vPBS and incubated for 2 min on ice. Cells were centrifuged for 5 min at 2000 × g, resuspended in vPBS, and settled on slides for 10 min. Slides were incubated for 30 min in −20˚C methanol and rehydrated for 10 min in PBS (8-mg/ml NaCl, 0.2-mg/ml KCl, 1.44-mg/ml Na_2_HPO_4_, 0.24-mg/ml KH_2_PO_4_). Cells were incubated for 1 h with primary antibodies diluted in PBS (rabbit anti-KIN-E 1:1,000; mouse mAb25 1:10 (Dacheux *et al.*, 2012); mouse L8C4 (PFR) 1:10 (Kohl *et al.*, 1999); mouse L3B2 (FAZ) 1:10 (Kohl *et al.*, 1999); and rabbit anti-BILBO1 1:4,000 [Esson *et al.*, 2012]). Cells were washed three times in PBS and incubated in a dark moist chamber for 1 h with secondary antibodies, all at a 1:200 dilution in PBS (anti-rabbit AF488 [ThermoFisher Scientific A11008], anti-rabbit AF564 [ThermoFisher Scientific A11036], anti-mouse AF488 [ThermoFisher Scientific A11001], anti-mouse AF568 [ThermoFisher Scientific A11004], anti-rat AF488 [ThermoFisher Scientific A21208]). Kinetoplasts and nuclei were stained with DAPI (10 μg/ml) for 4 min. Slides were mounted with ProLong Gold antifade reagent (ThermoFisher Scientific P36930). Images were acquired with a Zeiss AxioImager microscope, equipped with a Hamamatsu Orca 03 camera, using iVision software version 4.5.6r4 (BioVision Technologies), and analyzed with Fiji ImageJ version 1.51 or ImageJ2 version 1.54 (Schindelin *et al.*, 2012).

For DAPI staining of nuclei and kinetoplasts, parental and TbKIN-E^RNAi^-uninduced and -induced cells (at 0, 16, and 24 h after induction with 1-μg/ml tetracycline) in midlog phase were harvested and washed once in vPBS. The cells were fixed in 1% paraformaldehyde in vPBS for 2 min on ice. Cells were centrifuged 5 min at 2000 × g, resuspended in vPBS, and settled onto glass slides. Cells were permeabilized 30 min in −20˚C methanol, rehydrated with PBS, and stained with DAPI (1 μg/ml) for 3 min. Slides were mounted with ProLong Gold antifade. Images were acquired using a Zeiss AxioImager microscope and Hamamatsu Orca 03 camera with the same exposure time between samples, using iVision software version 4.5.6r4, and analyzed and quantified with Fiji ImageJ version 1.51 or ImageJ2 version 1.54.

### Live cell imaging

90-13 cells and TbKIN-E^RNAi^ were deposited onto glass bottom dishes 24-h following induction with 1-μg/ml tetracycline. Images were acquired at 20 frames/s with an Olympus IX71 microscope, equipped with an optiMOS™ sCMOS camera, using Micro-manager software (Edelstein *et al.*, 2014) and analyzed with Fiji ImageJ version 1.51 or ImageJ2 version 1.54.

### Electron microscopy

For TEM, 90-13 and TbKIN-E^RNAi^ cells induced for 16 or 24 h with 1-μg/ml tetracycline were collected in midlog phase and fixed for 30 min in HMI-9 with 2% glutaraldehyde at room temperature, and incubated overnight at 4°C. Cells were pelleted for 10 min at 1000 × *g*, resuspended in 2% very low gelling-point agarose (Sigma A5030) in water, pelleted again, and incubated for 15 min on ice. Agarose was cut into small pieces, rinsed twice in 0.1-M sodium cacodylate buffer (NaO_2_As(CH_3_)_2_), and incubated for 1 h in 1% osmium tetroxide (OsO_4_) in 0.1-M sodium cacodylate buffer with 1.6% potassium ferricyanide (pH 7.2). After three washes with 0.1-M sodium cacodylate buffer (pH 7.2), cells were dehydrated in successively higher concentrations of acetone (35%, 50%, 70%, 80%, 95%, 100%, and 100%) for 10-min incubations each. Cells were then incubated in acetone:resin (Eponate 12P) 2:1 for 30 min, 1:1 for 30 min and 1:2 for 1 h, followed by incubation in pure resin for 72 h. Cells were centrifuged for 10 min in a benchtop microfuge and moved into a new tube containing pure resin with accelerant benzyl dimethylamine (BDMA) for 30 min, and then for 4 h. Resin was left to polymerize overnight at 60°C in silicon molds or flat bottom capsules. Ultrathin sections were cut with an Ultracut E microtome (Reichert Jung) to ∼ 70-nm thick. Sections were loaded on formvar-coated mesh or slot grids (Electron Microscopy Sciences, G100-Cu, S2010-NOTCH) and stained with 2% uranyl acetate and lead citrate. Samples were visualized on Tecnai 12 transmission electron microscope with a UltraScan®1000XP CCD Camera and with the Gatan Digital micrograph software and processed with Fiji ImageJ version 1.51 or ImageJ2 version 1.54.

### Bioinformatic analysis

Distinct prediction software was used to identify domains within the KIN-E protein. The motor domain (aa 14–336) was identified using Pfam (Mistry *et al.*, 2021). The ARM domains (aa 480–653) were identified using SMART (Simple Modular Architecture Research Tool; Letunic and Bork, 2018). The CalpainIII-like domains (aa 713–997) were identified as in (An and Li, 2018) by alignment of the m-calpain domain III-like domains (mCL#1 and mCL#2) of KIN-E with the domain III of the human m-calpain protein (PBD code: 1KFU). The coiled-coil domain (aa 1171–1213) was identified using SMART.

### Statistical analysis

The statistical parameters and significance are reported in the figure legends. Statistical analyses were performed using GraphPad PRISM v.8.

## Abbreviations used:

BB: basal body
BSF: bloodstream form
FAZ: flagellum attachment zone
FP: flagellar pocket
FPC: flagellar pocket collar
GST: glutathione *S*-transferase
IFT: intraflagellar transport
kDNA: kinetoplast DNA
MtQ: microtubule quartet
PCF: procyclic trypomastigote or procyclic form
PFR: paraflagellar rod
TAC: tripartite attachment complex
TEM: transmission electron microscopy
VSG: variable surface glycoprotein
