# Supplementary material for: FAZ assembly in bloodstream form Trypanosoma brucei requires kinesin KIN-E
Source: Mol Biol Cell. 2023 Aug 17;34(10):ar103. doi: 10.1091/mbc.E23-01-0022 (PMC10551704; doi:10.1091/mbc.E23-01-0022)
Supplement: Supplementary file 5 [file mbc-34-ar103-s001.pdf]

Supplementary Materials

Molecular Biology of the Cell

Albisetti *et al*.

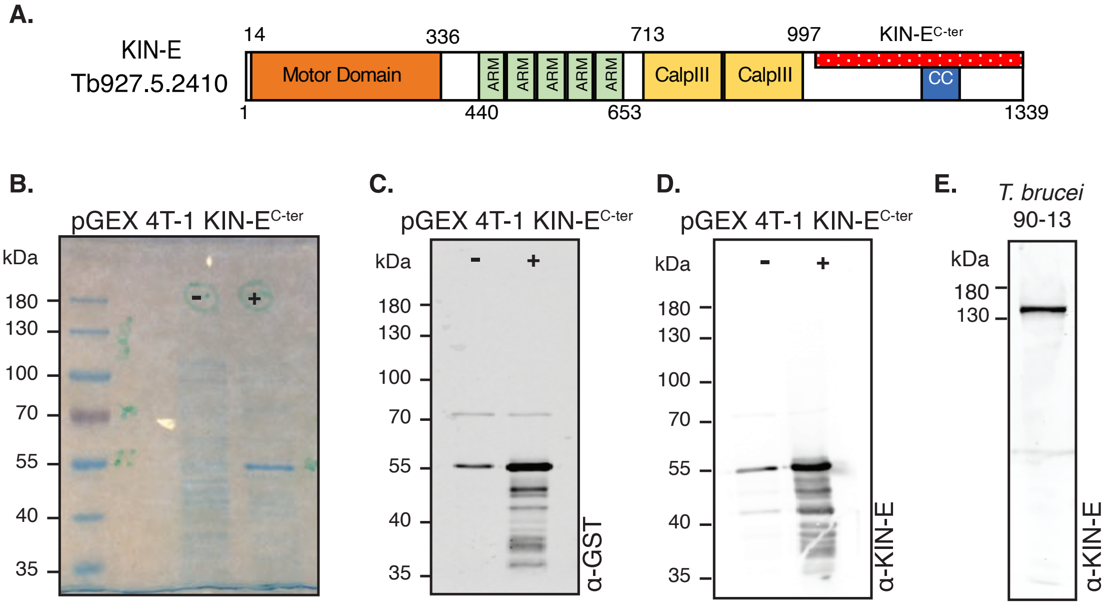

Figure S1 Specificity of the rabbit anti-KIN-E antibody tested by western blotting.
(A) Schematic representation of KIN-E (Tb927.5.2410), comprising the motor domain (orange), the armadillo repeats (ARM, green), the two CalpainIII-like domains (CalpIII, yellow), the short coiled-coil domain (CC, blue) and the KIN-E$^{C\text{-}ter}$ (1105-1339 aa, red with dots, used to raise an anti-KIN-E antibody). **(B)** SDS-PAGE gel, stained with SimpyBlue™ SafeStain, showing the expression of GST-KIN-E$^{C\text{-}ter}$ in 1 mM IPTG-induced (+) bacteria versus uninduced (-) bacteria. **(C,D)** Western blots of bacterial extracts (from $5x10^8$ cells in each lane) either uninduced or induced to express GST-KIN-E$^{C\text{-}ter}$ and probed with (C) anti-GST or (D) anti-KIN-E antibodies. **(E)** Western blot of *T. brucei* 90-13 whole cell lysate (from $5x10^6$ *T. brucei* cells) probed with anti-KIN-E antibody, showing a single band at around 150 kDa (KIN-E molecular mass 149 kDa).
