# Supplementary material for: FAZ assembly in bloodstream form Trypanosoma brucei requires kinesin KIN-E
Source: Mol Biol Cell. 2023 Aug 17;34(10):ar103. doi: 10.1091/mbc.E23-01-0022 (PMC10551704; doi:10.1091/mbc.E23-01-0022)
Supplement: Supplementary file 6 [file mbc-34-ar103-s002.pdf]

FAZ assembly in bloodstream form Trypanosoma brucei requires kinesin KIN-E

Anna Albisetti, Robert Douglas, and Matthew Welch

Corresponding author(s): Matthew Welch, University of California, Berkeley

Review Timeline:

Submission Date:	2023-01-25
Editorial Decision:	2023-05-06
Revision Received:	2023-07-17
Accepted:	2023-07-20

Editor-in-Chief: Thomas Pollard

Transaction Report:

(Note: With the exception of the correction of typographical or spelling errors that could be a source of ambiguity, letters and reports are not edited. The original formatting of letters and referee reports may not be reflected in this compilation.)

RE: Manuscript #E23-01-0022
TITLE: FAZ assembly in bloodstream form Trypanosoma brucei requires kinesin KIN-E

Dear Dr. Welch,

Thank you for submitting your manuscript, entitled "FAZ assembly in bloodstream form Trypanosoma brucei requires kinesin KIN-E", to MBoC. The manuscript was reviewed by two experts. While our reviewer #1 only requested minor modifications, reviewer #2 raised multiple concerns, including a possible relationship between KIN-E and the flagellar groove and a focus on direct effects of the KIN-E depletion. These concerns need to be properly addressed before the manuscript can be further considered for publication. We thus invite you to revise the manuscript and are looking forward to seeing the revised manuscript soon.

Xueliang Zhu
PhD, professor
Monitoring editor of MBoC

Dear Dr. Welch,

The review of your manuscript, referenced above, is now complete. The Monitoring Editor has decided that your manuscript is not acceptable for publication at this time, but may be deemed acceptable after specific revisions are made, as described in the Monitoring Editor's decision letter above and the reviewer comments below.

A reminder: Please do not contact the Monitoring Editor directly regarding your manuscript. If you have any questions regarding the review process or the decision, please contact the MBoC Editorial Office (mboc@ascb.org).

When submitting your revision include a rebuttal letter that details, point-by-point, how the Monitoring Editor's and reviewers' comments have been addressed. (The file type for this letter must be "rebuttal letter"; do not include your response to the Monitoring Editor and reviewers in a "cover letter.") Please bear in mind that your rebuttal letter will be published with your paper if it is accepted, unless you have opted out of publishing the review history.

Authors are allowed 90 days to submit a revision. If this time period is inadequate, please contact us at mboc@ascb.org.

Revised manuscripts are assigned to the original Monitoring Editor whenever possible. However, special circumstances may preclude this. Also, revised manuscripts are often sent out for re-review, usually to the original reviewers when possible. The Monitoring Editor may solicit additional reviews if it is deemed necessary to render a completely informed decision.

In preparing your revised manuscript, please follow the instruction in the Information for Authors (www.molbiolcell.org/info-for-authors). In particular, to prepare for the possible acceptance of your revised manuscript, submit final, publication-quality figures with your revision as described.

To submit the rebuttal letter, revised manuscript, and figures, use this link: Link Not Available

MBoC offers the option to publish your work with immediate open access. Open access can increase the discoverability and usability of your research. If you would like to publish your paper with immediate open access but did not select this option during initial submission, please contact the editorial office at mbc@ascb.org.

Thank you for submitting your manuscript to MBoC. We look forward to receiving your revised paper.

Sincerely,

Eric Baker
Journal Production Manager
MBoC Editorial Office
mbc@ascb.org

Reviewer #1 (Remarks to the Author):

Review:
In this paper the authors examine function of an orphan kinesin, KIN-E, in bloodstream stage Trypanosoma brucei. They find that KIN-E is localized to the flagellum and enriched at the tip of the flagellum. Using an RNAi knockdown strategy they find that depletion of KIN-E is lethal. Loss of KIN-E leads to a severe defect in the flagellum's lateral connection to the cell body along the flagellum attachment zone (FAZ). The authors use FAZ markers to show that loss of KIN-E disrupts assembly of the FAZ, explaining the flagellum attachment defect. The authors additionally show that KIN-E is required for localization of the flagellar protein FLAM3 to the flagellum tip. Similar results had been seen previously for procyclic (insect stage) cells. Video analysis indicates the detached flagellum retains the ability to beat, so flagellum assembly and movement are intact and the primary defect appears restricted to the FAZ. The KIN-E knockdown also exhibits defects in cytokinesis and segregation of the mitochondrial genome, the "kDNA", as well as vesicle accumulation at the flagellar pocket, which the authors attribute to endocytic defects based on ultrastructural analysis of the vesicles. The work is rigorous, well written, and presented well. While the KIN-E protein had been studied previously in procyclic (insect) stage parasites, with similar results, this paper is the first study in the infectious, bloodstream stage parasites, and the work extends earlier findings with discovery of a lethal phenotype and motility analysis of the flagellum. The importance of studying stage-specific functions of flagellar proteins is emphasized by recent work showing that some flagellar proteins exhibit stage-specific localization in T brucei, e.g. Bachmaier, S. et al. Nat Commun 13, 5445 (2022). While prior work has shown flagellar defects are associated with defective cytokinesis and kDNA segregation, the authors present one of the most detailed ultrastructural analyses of the kDNA defect and to my knowledge the first report of vesicle accumulation defect in flagellar mutants.

I have only a few suggestions for textual changes that might strengthen the manuscript.
1. Lines 224-226, describing FLAM3 localization in the KIN-E KD. I recommend rewording this so it doesn't sound like you are saying FLAM3 is localized to the tip of the new flagellum at 0hpi, 24hpi, and 48hpi.
2. Consider a diagram to illustrate the kinesin family of T brucei, emphasizing the "orphan kinesins" and what is know regarding their function in these organisms.
Include a reference to the Bachmaier 2022. Nat Commun 13, 5445 paper, illustrating the importance of stage-specific study of flagellar proteins.

Reviewer #2 (Remarks to the Author):

In this work, the function of the orphan kinesin KIN-E has been studied in the bloodstream form of Trypanosoma brucei. Previous work by the authors and other groups have established that the kinesin is essential for cell division and the maintenance of cellular morphology in the insect-resident procyclic form of the parasite. In the bloodstream form of the parasite, KIN-E localizes along the length of the flagellum and appears to be recruited to the tip of the new flagellum during cell division. KIN-E depletion blocks the formation of a new FAZ, leading to detached (but motile) new flagella, cytokinetic defects, and eventual cell death. There appears to be a subtle alteration in the endocytic/exocytic pathway that produces elevated levels of vesicles in proximity to the flagellar pocket. The flagellum-side FAZ component FLAM3 is mislocalized in KIN-E RNAi cells, which is consistent with the results in procyclics that showed that FLAM3 interacts with KIN-E and could be a potential cargo. Overall, this work shows that KIN-E is essential for the formation of the new FAZ, likely via the construction of the flagellum-side domain, and is another example of how FAZ defects have a severe impact on cytokinesis in T. brucei cells, likely by disrupting the assembly and positioning of the cleavage furrow.

The analysis of KIN-E in BSFs is very useful, as most of the orphan kinesins have only been analysed in PCFs. The observation of FP-adjacent vesicles is very interesting and could provide new insights into the consequences of inhibiting the construction of a new FAZ during cell division. I have several concerns about the work in its current form, both in terms how the work fits into our current thinking about trypanosome cell division and specifics about experiments were conducted, that should be addressed.

Major comments
1. Recently, a structure called the flagellar groove has been identified in BSFs that houses the tip of the new flagellum within the cell body during cell division. Both flagellar-side and cell-body side proteins have been identified as groove components, including several FAZ components. It seems very likely that KIN-E is a component of the groove, especially since FLAM3 was recently localized to the structure. The authors should put their work on KIN-E within this context, including citing Hughes et al., 2013 and Smithson et al., 2022 for previous work on the flagellar groove.

2. It is clear that KIN-E is essential for the formation of a new FAZ and that the lack of the new FAZ disrupts cell division. My main concern is that blocking FAZ assembly leads to a very broad range of defects in cytokinesis and other processes, including total blockage of cell division, aberrantly placed cleavage furrows that produce cells lacking nuclei or kinetoplasts, and even more complex secondary and tertiary effects that arise due to the initial defects as the cells continue to attempt to divide. This is especially true in asynchronized cells because they will likely lose KIN-E at different points of the cell cycle, which could lead to different phenotypic outcomes. The authors have included analysis of cells, such as Fig 4Bi, Fig 5Aii, Fig 3A (the cell shown),

that are likely to be the result of prolonged KIN-E depletion leading to additional defects that aren't directly attributable to KIN-E. Based off of the DNA counts in Fig 4, I think it would be very difficult to assign direct outcomes to KIN-E depletion at the 24 h point in the time course. I think many of the complex phenotypes observed are not directly attributable to KIN-E function. The authors should focus on the 16 h timepoint- earlier may have been better.

3. Are the authors using pleomorphs (90-13) for these experiments? If so, is the transition from slender to stumpy being accounted for as part of the experimental setup?

4. For the IFA images, it is recommended to change red/green co-labelling to magenta/green to make it easier for people with colour-blindness who might interact with the manuscript.

5. While the authors have been able to show that the 1K2N cells in their KIN-E RNAi likely arise due to a basal body segregation defect, there are significant populations of 1K0N and 0K1N cells that suggest that aberrant cell divisions are occurring. These cells combined are as abundant as the 1K2N cells. It seems likely that 1K0N and 0K1N cells are the product of aberrant cell divisions, rather than just an overall block in cell division. They should be accounted for in the discussion.

6. It is possible that bocking FAZ assembly disrupts the construction of a new flagellar pocket, as the structures at the top of the FP are closely associated the microtubule quartet and the initiation point of the new FAZ. Disrupting new FP assembly leads to the two flagella being confined within a single FP, which makes it impossible to properly segregate the kinetoplasts because the basal bodies are confined within a single structure.

Minor comments

Lines 48-51: In the BSF, the FP is not the only site devoid of subpellicular microtubules. See Hughes et al., 2013 for their work on the flagellar groove using 3D electron microscopy. The groove is also devoid of subpellicular microtubules and is a membrane invagination containing the distal tip of the growing new flagellum.

Line 50: There are newer FP references- Fields and Carrington Nature rev micro, Sunter Trends in Parasitology.

Line 56: I would choose a different word than "Extracellular"

Line 96: For non-experts, it might be good to explain the division cycle in T. brucei, explaining the retention of cell morphology and the fact that the new and old flagella are placed at specific points within the cell body. Otherwise going straight into "new and old flagellum" will be hard for non-tryp people to parse.

Line 97: "Occasion" should be "Occasional"

Line 168-175: I'm not sure I follow the logic here. BILBO labels the FPC, which is at the top of the pocket and is necessary for the assembly of the flagellar pocket. There are several examples of cells where there are defects in FPC/pocket duplication without any impact on kDNA duplication. TbPLK RNAi comes to mind- you can block production of a new pocket without affecting kDNA duplication. BILBO RNAi actually has no effect on kDNA duplication or partitioning. So I don't know what the authors are trying to say by looking at the Bilbo signal here? I would also say that the cell in Fig 5ii is very aberrant- it looks like it has failed one round of cell division and is attempting to initiate a second round. The things that are going on in this cell likely do not represent the most proximal effects of KIN-E depletion, but rather secondary or even tertiary effects. Assigning specific phenotypes at this point to KIN-E depletion is quite difficult.

Lines 177-189: This analysis is very clear, but considering that zoids (1K0N) and 0K1N cells are as common, if not more common than 1K2N cells, it is hard to specifically say that a lack of K separation is the most direct explanation of KIN-E function.

Lines 191-205: the EM here is very nice. Line 198: VSG is variant surface glycoprotein, singular- referring to the single VSG isoform expressed. While there is clearly some change in vesicular profiles here, it should be noted that BSFs are exquisitely sensitive to changes in endo/exocytosis- see Clathrin RNAi, MORN RNAi, Actin RNAi. If there was some global dysregulation of endo/exocytosis, you would expect to see a bigeye phenotype with swelling of the FP and rapid cell lysis. Could the accumulation of vesicles have something to do with the delivery of FAZ material to the pocket? Since the cell-body side FAZ is not being assembled, I wonder if these vesicles may contain those components. If these vesicles represent a subpopulation tasked with trafficking FAZ components, it could help explain the phenotype and why you are not seeing the catastrophic consequences of disrupting the bulk endo/exocytosis.

Figure 1

It appears that KIN-E localizes to the flagellar groove (Hughes et al., 2013; Smithson et al., 2022). Would recommend mentioning this or even doing a colocalization IF with known groove proteins (FAZ10/DOT1, FAZ15, FCP7).

I think it's difficult to have FAZ/PFR/axoneme as the same color in a single figure panel. It's very difficult to distinguish the structures since they all look very similar. I'd consider separating the panel based on the KIN-E colabel, or use different colors for mAb24, PFR, and FAZ. I'm not sure that the description of the KIN-E signal in text matches what I observe in the figure. Based off of the images, I would say that the KIN-E signal is along both old and new flagella, with concentration at the flagellum tip in early cell cycle stage cells. Once there's a new flagellum, the KIN-E signal seems to relocate onto the tip of the new flagellum. This might suggest that KIN-E relocalizes to the new flagellum as it grows, which might point to function. The image in D does not highlight the signal along the old and new flagella- it is not clear that there's an offset, especially since the the inset shows the distal new flagellum tip labeling and not the signal along the cell body, where you'd expect to see the offset? For A and B, the cells look to be at quite different magnifications- I do not think the scale bars could be the same for both images.

Figure legend - Line 489: I think the authors meant L8C4 instead of L3B2.

Figure legend - Line 491-494: Would recommend not using the nomenclature "anterior" to describe the flagellum. It would be better to stick with distal/proximal to keep consistent with the manuscript and other literature.

Figure 2Ci: I think this cell is actually fairly abnormal. The new flagellum has been released from the groove but there's no evidence of a cleavage furrow. My guess is that the new FAZ isn't long enough.

Figure 3:
Figure 3 and line 132: In the figure, the category is called "unattached flagella" but in the manuscript the category is "detached flagella". Would recommend changing the figure category to match with the manuscript.

Figure 3A: The image shown here is a little confusing- the authors have been suggesting that the new flagellum is specifically impacted by KIN-E depletion. Based off of cell cycle stage and the length of the flagellum, it seems like the detached flagellum here is the only flagellum in this cell, which is the "old" flagellum. This would suggest that the old FAZ/Flagellum are being impacted by KIN-E depletion, which is not consistent with the hypotheses and data in the rest of the paper. I'd consider replacing the image or revising what the authors think the outcome of KIN-E depletion is.

Fig 3B: are the authors proposing that the KIN-E signal along the old and new flagellum decline first, leaving behind a small puncta where the new flagellum detaches from the cell surface? Because considering the other images in the paper, KIN-E accumulates at the tip of the new flagellum as it extends. It seems odd that it would relocate to this position, especially since the tip of the new FAZ and the new flagellum are offset from one another?

Figure 4:

Figure 4A: Considering you have the table with the numerical percentages relating to the graph, I would consider condensing your categories for the "abnormal+/-df" part of the graph. Perhaps grouping the 1K0N, 0K1N, ?K?N and No DNA into one category would make it more reader friendly. Anyone who is interested in more detail can look to the table for clarification.

Figure 5Aii. Very difficult to draw conclusions from second cell because it's undergone multiple rounds of abortive/aberrant division- what are the primary effects vs. later ones?

Fig 7B. While it is apparent that KIN-E RNAi affects the localization of FLAM3, the fact that KIN-E RNAi blocks the formation of a new FAZ entirely makes it hard to know if the loss of FLAM3 is specific, or is just a consequence of the new FAZ not being built.

Typos/Corrections
• Line 29: Nagana should be italicized.
• Line 38: Remove "form" after bloodstream trypomastigote.
• Line 45/54: Via is italicized in one line but not the other. Make consistent.
• Line 92: Add a comma after western blotting.
• Line 96: Rephrase sentence as "...lengths of the old and new...".
• Line 97: Change "Occasion" to "Occasional".
• Line 99: clarify sentence by adding in "distal" into "KIN-E signal was seen at the tips of both short...".
• Line 109: The statement "KIN-E and the FAZ followed parallel paths but did not overlap" needs a clarification sentence saying that KIN-E and FAZ1 (L3B2) likely localize to different domains of the FAZ. FAZ1 is on the cell body side but later on the authors colocalize KIN-E with FLAM3, on the flagellum side of the FAZ.
• Line 121: Ensure that figure nomenclature is consistent through manuscript (Fig. 2B) here but (Fig 2A) previously.
• Line 170: Remover "in" after "24 hpi).
• Line 216: FLAM3 has already been localized in BSFs (Smithson et al., 2022). It localizes to the groove.
• Line 227: Remove "...in flagella" to make the sentence flow better.

Editor's comments:
"While our reviewer #1 only requested minor modifications, reviewer #2 raised multiple concerns, including a possible relationship between KIN-E and the flagellar groove and a focus on direct effects of the KIN-E depletion. These concerns need to be properly addressed before the manuscript can be further considered for publication. We thus invite you to revise the manuscript and are looking forward to seeing the revised manuscript soon."

Response: We have addressed the reviewer's comments as noted below.

Reviewer #1 (Remarks to the Author):
"In this paper the authors examine function of an orphan kinesin, KIN-E, in bloodstream stage Trypanosoma brucei. They find that KIN-E is localized to the flagellum and enriched at the tip of the flagellum. Using an RNAi knockdown strategy they find that depletion of KIN-E is lethal. Loss of KIN-E leads to a severe defect in the flagellum's lateral connection to the cell body along the flagellum attachment zone (FAZ). The authors use FAZ markers to show that loss of KIN-E disrupts assembly of the FAZ, explaining the flagellum attachment defect. The authors additionally show that KIN-E is required for localization of the flagellar protein FLAM3 to the flagellum tip. Similar results had been seen previously for procyclic (insect stage) cells. Video analysis indicates the detached flagellum retains the ability to beat, so flagellum assembly and movement are intact and the primary defect appears restricted to the FAZ. The KIN-E knockdown also exhibits defects in cytokinesis and segregation of the mitochondrial genome, the "kDNA", as well as vesicle accumulation at the flagellar pocket, which the authors attribute to endocytic defects based on ultrastructural analysis of the vesicles. The work is rigorous, well written, and presented well. While the KIN-E protein had been studied previously in procyclic (insect) stage parasites, with similar results, this paper is the first study in the infectious, bloodstream stage parasites, and the work extends earlier findings with discovery of a lethal phenotype and motility analysis of the flagellum. The importance of studying stage-specific functions of flagellar proteins is emphasized by recent work showing that some flagellar proteins exhibit stage-specific localization in T brucei, e.g. Bachmaier, S. et al. Nat Commun 13, 5445 (2022). While prior work has shown flagellar defects are associated with defective cytokinesis and kDNA segregation, the authors present one of the most detailed ultrastructural analyses of the kDNA defect and to my knowledge the first report of vesicle accumulation defect in flagellar mutants."

Response: We thank the reviewer for this positive assessment of our work.

"I have only a few suggestions for textual changes that might strengthen the manuscript.
1. Lines 224-226, describing FLAM3 localization in the KIN-E KD. I recommend rewording this so it doesn't sound like you are saying FLAM3 is localized to the tip of the new flagellum at 0hpi, 24hpi, and 48hpi."

Response: We thank the reviewer for spotting this unclear sentence. To improve clarity, we split this into two sentences on lines 256-258, as follows: "FLAM3 was at the tip of new flagella at 0 hpi. However, at 24 hpi and 48 hpi, FLAM3 was confined to the proximal end of new flagella (Fig 7B), between the kDNA and the origin of the PFR."

"2. Consider a diagram to illustrate the kinesin family of T brucei, emphasizing the "orphan kinesins" and what is known regarding their function in these organisms."

Response: We thank the reviewer for this suggestion. Such diagrams can be found in the publications cited in the Introduction on line 100-101 (Wickstead and Gull, 2006; Wickstead *et al.*, 2010). For this reason, and because our manuscript does not focus on kinesin phylogeny, we have elected not to include a diagram in our paper.

"3. Include a reference to the Bachmaier 2022. Nat Commun 13, 5445 paper, illustrating the importance of stage-specific study of flagellar proteins."

Response: We now add a sentence to the Introduction on line 104-106 that references this paper and states: "Although the importance of stage-specific studies of flagellar protein function has recently been emphasized (Bachmaier *et al.*, 2022), the role of KIN-E in the medically relevant BSF cells has remained undetermined."

Reviewer #2 (Remarks to the Author):
"*In this work, the function of the orphan kinesin KIN-E has been studied in the bloodstream form of Trypanosoma brucei. Previous work by the authors and other groups have established that the kinesin is essential for cell division and the maintenance of cellular morphology in the insect-resident procyclic form of the parasite. In the bloodstream form of the parasite, KIN-E localizes along the length of the flagellum and appears to be recruited to the tip of the new flagellum during cell division. KIN-E depletion blocks the formation of a new FAZ, leading to detached (but motile) new flagella, cytokinetic defects, and eventual cell death. There appears to be a subtle alteration in the endocytic/exocytic pathway that produces elevated levels of vesicles in proximity to the flagellar pocket. The flagellum-side FAZ component FLAM3 is mislocalized in KIN-E RNAi cells, which is consistent with the results in procyclics that showed that FLAM3 interacts with KIN-E and could be a potential cargo. Overall, this work shows that KIN-E is essential for the formation of the new FAZ, likely via the construction of the flagellum-side domain, and is another example of how FAZ defects have a severe impact on cytokinesis in T. brucei cells, likely by disrupting the assembly and positioning of the cleavage furrow.*

The analysis of KIN-E in BSFs is very useful, as most of the orphan kinesins have only been analysed in PCFs. The observation of FP-adjacent vesicles is very interesting and could provide new insights into the consequences of inhibiting the construction of a new FAZ during cell division. I have several concerns about the work in its current form, both in terms how the work fits into our current thinking about trypanosome cell division and specifics about experiments were conducted, that should be addressed."

Response: We thank the reviewer for their positive tone and constructive suggestions. Each specific suggestion is addressed below.

"Major comments
1. Recently, a structure called the flagellar groove has been identified in BSFs that houses the tip of the new flagellum within the cell body during cell division. Both flagellar-side and cell-body side proteins have been identified as groove components, including several FAZ components. It seems very likely that KIN-E is a component of the groove, especially since FLAM3 was recently localized to the structure. The authors should put their work on KIN-E within this context, including citing Hughes et al., 2013 and Smithson et al., 2022 for previous work on the flagellar groove."

Response: We thank the reviewer for this insight and suggestion. To address this point, we have added the following sentence to the Introduction on line 69-73: “During the cell division cycle, a new flagellum is assembled from the BB and, as it grows, its distal tip is tethered to the old flagellum. In PCF cells, the tethering structure is called the flagellar connector (Moreira-Leite *et al.*, 2001; Briggs *et al.*, 2004; Davidge *et al.*, 2006), whereas in BSF cells it is called the flagellar groove (Hughes *et al.*, 2013; Smithson *et al.*, 2022).” Moreover, we mention this in the Results section on line 127-130: “KIN-E signal was seen at the distal tips of both short (Figure 1B) and long (Figure 1C) new flagella, suggesting it selectively tracks the growing flagellum tip in the flagellar groove (Hughes *et al.*, 2013; Smithson *et al.*, 2022).”

“2. It is clear that KIN-E is essential for the formation of a new FAZ and that the lack of the new FAZ disrupts cell division. My main concern is that blocking FAZ assembly leads to a very broad range of defects in cytokinesis and other processes, including total blockage of cell division, aberrantly placed cleavage furrows that produce cells lacking nuclei or kinetoplasts, and even more complex secondary and tertiary effects that arise due to the initial defects as the cells continue to attempt to divide. This is especially true in asynchronized cells because they will likely lose KIN-E at different points of the cell cycle, which could lead to different phenotypic outcomes. The authors have included analysis of cells, such as Fig 4Bi, Fig 5Aii, Fig 3A (the cell shown), that are likely to be the result of prolonged KIN-E depletion leading to additional defects that aren't directly attributable to KIN-E. Based off of the DNA counts in Fig 4, I think it would be very difficult to assign direct outcomes to KIN-E depletion at the 24 h point in the time course. I think many of the complex phenotypes observed are not directly attributable to KIN-E function. The authors should focus on the 16 h timepoint- earlier may have been better.”

Response: We agree that the primary defect caused by KIN-E depletion is impaired FAZ assembly and that the failure to assemble the FAZ likely leads to a complex spectrum of other defects which include disruption of cell division processes. To clarify this point, we have changed the section headings to describe the observed defects caused by KIN-E depletion, rather than appearing to imply a direct role for KIN-E in cell division. For example, rather than the heading “KIN-E is critical for cytokinesis,” we now use the heading “KIN-E depletion disrupts cell division.” Similarly, rather than the heading “KIN-E influences kDNA segregation,” we now use the heading “KIN-E depletion impacts kDNA segregation.” We have also changed the conclusion sentences to simply summarize the observations we are reporting.

“3. Are the authors using pleomorphs (90-13) for these experiments? If so, is the transition from slender to stumpy being accounted for as part of the experimental setup?”

Response: Yes, 90-13 is a pleomorphic strain. However, we never grow cells to high enough density to transition from the slender to the stumpy form.

“4. For the IFA images, it is recommended to change red/green co-labelling to magenta/green to make it easier for people with colour-blindness who might interact with the manuscript.”

Response: We have taken this under advisement for future publications but prefer not to have to remake all of the figures for this publication.

"5. While the authors have been able to show that the 1K2N cells in their KIN-E RNAi likely arise due to a basal body segregation defect, there are significant populations of 1K0N and 0K1N cells that suggest that aberrant cell divisions are occurring. These cells combined are as abundant as the 1K2N cells. It seems likely that 1K0N and 0K1N cells are the product of aberrant cell divisions, rather than just an overall block in cell division. They should be accounted for in the discussion."

Response: We have modified line 310-311 in the discussion to read, "We observed a second major phenotype upon KIN-E depletion in BSF cells, which is the accumulation of multinucleated and multi-flagellated cells, as well as a small population of zoids, indicative of failure in cell division or aberrant cell division."

"6. It is possible that blocking FAZ assembly disrupts the construction of a new flagellar pocket, as the structures at the top of the FP are closely associated the microtubule quartet and the initiation point of the new FAZ. Disrupting new FP assembly leads to the two flagella being confined within a single FP, which makes it impossible to properly segregate the kinetoplasts because the basal bodies are confined within a single structure."

Response: This idea is potentially interesting. However, we are unaware of any published literature showing that blocking FAZ assembly disrupts the construction of the new FP. Moreover, the morphology of the FP does not seem to be affected by TEM. For this reason, we do not favor altering the text of the manuscript to address this point.

"Minor comments
Lines 48-51: In the BSF, the FP is not the only site devoid of subpellicular microtubules. See Hughes et al., 2013 for their work on the flagellar groove using 3D electron microscopy. The groove is also devoid of subpellicular microtubules and is a membrane invagination containing the distal tip of the growing new flagellum."

Response: As noted above in the response to this reviewer's major comment 2, we now mention the flagellar groove and cite Hughes *et al*. and Smithson *et al*.

"Line 50: There are newer FP references- Fields and Carrington Nature rev micro, Sunter Trends in Parasitology."

Response: We added the Field and Carrington reference on line 67, as suggested. The Sunter et al. reference is cited in the following paragraph on line 87.

"Line 56: I would choose a different word than "Extracellular""

Response: As suggested, we removed the phrase "on the extracellular side." The sentence on line 81-82 now reads "Transmembrane proteins including FLA1 and FLA1BP connect the cell body and flagellar membranes."

"Line 96: For non-experts, it might be good to explain the division cycle in T. brucei, explaining the retention of cell morphology and the fact that the new and old flagella are placed at specific points within the cell body. Otherwise going straight into "new and old flagellum" will be hard for non-tryp people to parse."

Response: As suggested, we have added a sentence to the Introduction on line 69-73 describing new flagellum assembly during the cell cycle.

"Line 97: "Occasion" should be "Occasional""

Response: Corrected on line 126 as also noted below.

"Line 168-175: I'm not sure I follow the logic here. BILBO labels the FPC, which is at the top of the pocket and is necessary for the assembly of the flagellar pocket. There are several examples of cells where there are defects in FPC/pocket duplication without any impact on kDNA duplication. TbPLK RNAi comes to mind- you can block production of a new pocket without affecting kDNA duplication. BILBO RNAi actually has no effect on kDNA duplication or partitioning. So I don't know what the authors are trying to say by looking at the Bilbo signal here? I would also say that the cell in Fig 5ii is very aberrant- it looks like it has failed one round of cell division and is attempting to initiate a second round. The things that are going on in this cell likely do not represent the most proximal effects of KIN-E depletion, but rather secondary or even tertiary effects. Assigning specific phenotypes at this point to KIN-E depletion is quite difficult."

Response: Our point for including the BILBO staining in Figure 5A is to show that KIN-E depleted cells are able to form a new flagellum, a new FP, and a new FPC. We then show in Figure 5B, 5C, and 5D that KIN-E depleted cells can also replicate their kDNA but not segregate it. This supports the conclusion that KIN-E depletion impacts kDNA segregation. As noted above in response to this reviewer's major comment 2, we agree that the kDNA segregation is likely a secondary consequence of FAZ disruption, and we now explicitly state this in the Discussion on line 331-332.

"Lines 177-189: This analysis is very clear, but considering that zoids (1K0N) and 0K1N cells are as common, if not more common than 1K2N cells, it is hard to specifically say that a lack of K separation is the most direct explanation of KIN-E function."

Response: We agree that the kDNA segregation defect may not reflect a direct role for KIN-E in this process. To clarify this, on line 331-332 we now state, "Failure in kDNA segregation may therefore be a secondary consequence of impaired FAZ formation."

"Lines 191-205: the EM here is very nice. Line 198: VSG is variant surface glycoprotein, singular- referring to the single VSG isoform expressed. While there is clearly some change in vesicular profiles here, it should be noted that BSFs are exquisitely sensitive to changes in endo/exocytosis- see Clathrin RNAi, MORN RNAi, Actin RNAi. If there was some global dysregulation of endo/exocytosis, you would expect to see a bigeye phenotype with swelling of the FP and rapid cell lysis. Could the accumulation of vesicles have something to do with the delivery of FAZ material to the pocket? Since the cell-body side FAZ is not being assembled, I wonder if these vesicles may contain those components. If these vesicles represent a subpopulation tasked with trafficking FAZ components, it could help explain the phenotype and why you are not seeing the catastrophic consequences of disrupting the bulk endo/exocytosis."

Response: We thank the reviewer for their appreciation of the TEM pictures. For the definition of VSG, we have removed the “s” so the text on line 49-50 now reads “variable surface glycoprotein (VSG).” We agree that the fact we do not see a bigeye phenotype means there is no global dysregulation of endocytosis. The explanation the reviewer gives is interesting. We now mention this in the discussion on lines 334-339, which now reads: “Vesicle accumulation is not accompanied by an expansion of the FP, as occurs following depletion of proteins important for protein entry into or endocytosis within the FP (Allen *et al.*, 2003; García-Salcedo *et al.*, 2004; Morriswood and Schmidt, 2015), suggesting that there is no global inhibition of endocytosis. The accumulated vesicles may be endocytic vesicles that have shed their clathrin coat, or exocytic vesicles that contain cell-body-side FAZ components that could not be assembled due to KIN-E deletion.”

“Figure 1
It appears that KIN-E localizes to the flagellar groove (Hughes et al., 2013; Smithson et al., 2022). Would recommend mentioning this or even doing a colocalization IF with known groove proteins (FAZ10/DOT1, FAZ15, FCP7).”

Response: We have altered the text on lines 127-130 to read, “KIN-E signal was seen at the distal tips of both short (Figure 1B) and long (Figure 1C) new flagella, suggesting it selectively tracks the growing flagellum tip in the flagellar groove (Hughes *et al.*, 2013; Smithson *et al.*, 2022).”

“I think it's difficult to have FAZ/PFR/axoneme as the same color in a single figure panel. It's very difficult to distinguish the structures since they all look very similar. I'd consider separating the panel based on the KIN-E colabel, or use different colors for mAb24, PFR, and FAZ. I'm not sure that the description of the KIN-E signal in text matches what I observe in the figure. Based off of the images, I would say that the KIN-E signal is along both old and new flagella, with concentration at the flagellum tip in early cell cycle stage cells. Once there's a new flagellum, the KIN-E signal seems to relocate onto the tip of the new flagellum. This might suggest that KIN-E relocalizes to the new flagellum as it grows, which might point to function. The image in D does not highlight the signal along the old and new flagella- it is not clear that there's an offset, especially since the the inset shows the distal new flagellum tip labeling and not the signal along the cell body, where you'd expect to see the offset? For A and B, the cells look to be at quite different magnifications- I do not think the scale bars could be the same for both images.”

Response: We thank the reviewer for these suggestions. To minimize confusion, we have elected to add a bit more space between panels A, B & C, and D. Moreover, we have added a new image to Figure 1D that more clearly highlights the offset between the KIN-E signal and the FAZ signal. Moreover, we have corrected the magnification and scale bars in the images to address the concern about different cell sizes and magnifications.

“Figure legend - Line 489: I think the authors meant L8C4 instead of L3B2.”

Response: We thank the reviewer for spotting this mistake! This correction has been made.

“Figure legend - Line 491-494: Would recommend not using the nomenclature "anterior" to describe the flagellum. It would be better to stick with distal/proximal to keep consistent with the manuscript and other literature.”

Response: Thank you for the suggestion. We have substituted “distal” for “anterior.”

“Figure 2Ci: I think this cell is actually fairly abnormal. The new flagellum has been released from the groove but there's no evidence of a cleavage furrow. My guess is that the new FAZ isn't long enough.”

Response: We appreciate this observation. However, the purpose of this image is point out the loss of KIN-E signal on the flagella along with the detached new flagellum, which we feel that the image effectively illustrates.

“Figure 3:
Figure 3 and line 132: In the figure, the category is called "unattached flagella" but in the manuscript the category is "detached flagella". Would recommend changing the figure category to match with the manuscript.”

Response: We have changed “unattached” to “detached” in Fig 3A.

“Figure 3A: The image shown here is a little confusing- the authors have been suggesting that the new flagellum is specifically impacted by KIN-E depletion. Based off of cell cycle stage and the length of the flagellum, it seems like the detached flagellum here is the only flagellum in this cell, which is the "old" flagellum. This would suggest that the old FAZ/Flagellum are being impacted by KIN-E depletion, which is not consistent with the hypotheses and data in the rest of the paper. I'd consider replacing the image or revising what the authors think the outcome of KIN-E depletion is.”

Response: Because the reviewer found the image in Figure 3A confusing, and because similar images are found in Figure 3B, we have removed the image from Figure 3A.

“Fig 3B: are the authors proposing that the KIN-E signal along the old and new flagellum decline first, leaving behind a small puncta where the new flagellum detaches from the cell surface? Because considering the other images in the paper, KIN-E accumulates at the tip of the new flagellum as it extends. It seems odd that it would relocate to this position, especially since the tip of the new FAZ and the new flagellum are offset from one another?”

Response: The main point we are making is that RNAi diminishes KIN-E signal on the new flagellum, corresponding with the depletion of KIN-E protein by western blotting. At the 16 h timepoint, we sometimes see residual KIN-E staining at the flagellum tip or at the site at which the new flagellum detaches from the cell body. It also seems unlikely to us that it is relocating to this position. Rather, residual KIN-E may remain at this position in some cases.

“Figure 4:
Figure 4A: Considering you have the table with the numerical percentages relating to the graph, I would consider condensing your categories for the "abnormal+/-df" part of the graph. Perhaps grouping the 1K0N, 0K1N, ?K?N and No DNA into one category would make it more reader friendly. Anyone who is interested in more detail can look to the table for clarification.”

Response: We thank the reviewer for the suggestion. We have condensed these phenotypes into one category in the graph.

“Figure 5Aii. Very difficult to draw conclusions from second cell because it's undergone multiple rounds of abortive/aberrant division- what are the primary effects vs. later ones?”

Response: As noted above in relation to Figure 5A, we agree that the kDNA segregation defect may not reflect a direct role for KIN-E in this process. To clarify this, on line 331-332 we now state, “Failure in kDNA segregation may therefore be a secondary consequence of impaired FAZ formation.”

“Fig 7B. While it is apparent that KIN-E RNAi affects the localization of FLAM3, the fact that KIN-E RNAi blocks the formation of a new FAZ entirely makes it hard to know if the loss of FLAM3 is specific, or is just a consequence of the new FAZ not being built.”

Response: FLAM3 has been shown to interact with KIN-E in PCF cells (An and Li, 2018), and we propose that the same is true in BSF cells.

“*Typos/Corrections*
- *Line 29: Nagana should be italicized.*
- *Line 38: Remove "form" after bloodstream trypomastigote.*
- *Line 45/54: Via is italicized in one line but not the other. Make consistent.*
- *Line 92: Add a comma after western blotting.*
- *Line 96: Rephrase sentence as "...lengths of the old and new...".*
- *Line 97: Change "Occasion" to "Occasional".*
- *Line 99: clarify sentence by adding in "distal" into "KIN-E signal was seen at the tips of both short...".*
- *Line 109: The statement "KIN-E and the FAZ followed parallel paths but did not overlap" needs a clarification sentence saying that KIN-E and FAZ1 (L3B2) likely localize to different domains of the FAZ. FAZ1 is on the cell body side but later on the authors colocalize KIN-E with FLAM3, on the flagellum side of the FAZ.*
- *Line 121: Ensure that figure nomenclature is consistent through manuscript (Fig. 2B) here but (Fig 2A) previously.*
- *Line 170: Remover "in" after "24 hpi).*
- *Line 216: FLAM3 has already been localized in BSFs (Smithson et al., 2022). It localizes to the groove.*
- *Line 227: Remove "...in flagella" to make the sentence flow better.”*

Response: We thank the reviewer for their careful proofreading! All of these corrections have been made.

RE: Manuscript #E23-01-0022R
TITLE: "FAZ assembly in bloodstream form Trypanosoma brucei requires kinesin KIN-E"

Dear Dr. Welch:

Thank you for sending back your revised manuscript. After careful examinations on the rebuttal letter and the changes in the manuscript, I believe that you have fully addressed the concerns of the reviewers and that it is no longer required to send the manuscript to them for a re-review. Therefore, I am pleased to accept your manuscript for publication in Molecular Biology of the Cell. Congratulations!

I agree that this manuscript is a good candidate for the special issue on the "Cell Biology of Organelles in Unicellular Parasites" and will leave the decision to the lead editors of the issue.

Sincerely,
Xueliang Zhu
Monitoring Editor
Molecular Biology of the Cell

Dear Dr. Welch:

Congratulations on the acceptance of your manuscript! Thank you for choosing to publish your work in Molecular Biology of the Cell (MBoC).

Within 10 days, an unedited PDF of your manuscript will be published on MBoC in Press, an early release version of the journal. The date your manuscript appears on this site is the official publication date.

Your copyedited and typeset manuscript will be scheduled for publication in the next available issue of MBoC. Our production team will notify you when the page proofs of your paper are ready for your review.

MBoC offers the option to publish your paper with immediate open access. Open access can increase the discoverability and usability of your research. If you would like to publish your paper with immediate open access but did not select this option during initial submission, please contact the MBoC Editorial Office (mbc@ascb.org).

We invite you to submit images related to your accepted manuscript to be considered for the journal cover. Please contact mboc@ascb.org to learn how to submit candidate cover images.

Authors of Articles and Brief Communications are also encouraged to create a short video abstract to accompany their article when it is published. These video abstracts, known as Science Sketches, are up to 2 minutes long and will be published on YouTube and then embedded in the article abstract. Science Sketch Editors on the MBoC Editorial Board will provide guidance as you prepare your video. Information about how to prepare and submit a video abstract is available at www.molbiolcell.org/science-sketches. Please contact mboc@ascb.org if you are interested in creating a Science Sketch.

We look forward to publishing your paper in MBoC.
Sign up for MBoC's new content alerts here https://tinyurl.com/3bcsyk3e

Sincerely,

Eric Baker
Journal Production Manager
MBoC Editorial Office
mbc@ascb.org
